# Music As a Sacred Cue? Effects of Religious Music on Moral Behavior

**DOI:** 10.3389/fpsyg.2016.00814

**Published:** 2016-06-07

**Authors:** Martin Lang, Panagiotis Mitkidis, Radek Kundt, Aaron Nichols, Lenka Krajčíková, Dimitris Xygalatas

**Affiliations:** ^1^Department of Anthropology, University of ConnecticutStorrs, CT, USA; ^2^LEVYNA Laboratory for the Experimental Research of Religion, Department for the Study of Religions, Masaryk UniversityBrno, Czech Republic; ^3^Center for Advanced Hindsight, Social Science Research Institute, Duke UniversityDurham, NC, USA; ^4^Interacting Minds Centre, Department of Culture and Society, Aarhus UniversityAarhus, Denmark; ^5^Interdisciplinary Centre for Organizational Architecture, Department of Management, Aarhus UniversityAarhus, Denmark; ^6^Department of Psychology, Faculty of Arts, Masaryk UniversityBrno, Czech Republic

**Keywords:** religion, music, associative learning, morality, priming

## Abstract

Religion can have an important influence in moral decision-making, and religious reminders may deter people from unethical behavior. Previous research indicated that religious contexts may increase prosocial behavior and reduce cheating. However, the perceptual-behavioral link between religious contexts and decision-making lacks thorough scientific understanding. This study adds to the current literature by testing the effects of purely audial religious symbols (instrumental music) on moral behavior across three different sites: Mauritius, the Czech Republic, and the USA. Participants were exposed to one of three kinds of auditory stimuli (religious, secular, or white noise), and subsequently were given a chance to dishonestly report on solved mathematical equations in order to increase their monetary reward. The results showed cross-cultural differences in the effects of religious music on moral behavior, as well as a significant interaction between condition and religiosity across all sites, suggesting that religious participants were more influenced by the auditory religious stimuli than non-religious participants. We propose that religious music can function as a subtle cue associated with moral standards via cultural socialization and ritual participation. Such associative learning can charge music with specific meanings and create sacred cues that influence normative behavior. Our findings provide preliminary support for this view, which we hope further research will investigate more closely.

## Introduction

Much psychological research conducted over the past decade has attempted to further scientific understanding of morality and ethical behavior by observing how environmental cues can enhance or degrade ethical behavior (Shariff and Norenzayan, 2007; Mead et al., 2009; Mazar and Zhong, 2010; John et al., 2014). Inferred social norms (Gino et al., 2009), ethical reminders (Mazar et al., 2008), and even decorative objects in a room (Krátký et al., 2016), have all been observed to affect dishonest behavior. This evidence suggests that automaticity plays an important role in moral decision-making based on perceptual cues (Bargh et al., 2012; Newell and Shanks, 2014). Making internalized norms salient via contextual cues can push people toward normative behavioral strategies (Cialdini et al., 1990; Hirsh et al., 2011), often without a conscious link between the two (Bargh and Morsella, 2008). As such, behavioral responses to moral dilemmas might result from the interplay between individual norms and contextual percepts, especially in a structured environment that is regulated by normative institutions (Graham et al., 2012).

A prime example of such a normative institution is religion. Religions often strongly impact the individual's socialization process, and through the use of reminders such as symbols and repeated rituals make group-specific norms salient (Durkheim, 1912; Norenzayan and Shariff, 2008; Xygalatas, 2013). Research in recent years has shown that religious situational factors enhance the saliency of norms and play a significant role in moral decision-making (for an extensive meta-analysis see Shariff et al., 2016). However, despite an ample body of research on religious prosociality, the effects of religious contextual cues on unethical behavior are less well-documented. Only a handful of studies have looked at the effects of religious cues on deterring cheating (Bering et al., 2005; Randolph-Seng and Nielsen, 2007; Mazar et al., 2008; Piazza et al., 2011). For example, Mazar et al. (2008) found lower cheating rates amongst participants who were asked to recall the 10 Commandments compared to those who had to recall 10 book titles. Similar results were observed when using other environmental cues, such as the Islamic call to prayer (Aveyard, 2014).

These studies suggest that people modify their decisions in response to sacred cues, similarly to the way they respond to other environmental cues (for instance light in the room—Zhong et al., 2010), and that religious environments might have complex effects on people's social behavior. However, the exact mechanisms underlying the perceptual-behavioral links that affect decision-making under the influence of sacred cues are still not fully understood. Researchers have traditionally primed concepts of spirituality implicitly through the use of religiously infused anagrams (Srull and Wyer, 1979). For example, “dessert divine was fork the” would be unscrambled by participants to “the dessert was divine” (Shariff and Norenzayan, 2007). Such priming can carry semantic associations with moral norms and might also invoke fear of supernatural punishment thereby inhibiting immoral behavior. Similarly, anthropomorphic depictions of eyes might evoke a feeling of being observed and trigger reputational concerns (Bateson et al., 2006; Krátký et al., 2016). But would the same effects on moral behavior hold for arbitrary stimuli associated with religion, for instance, specific objects, gestures, or music? While the meanings of words are formed during the process of early socialization, and associations with specific actions are reinforced by everyday use, religious symbols are often confined to specific domains of one's life. Their tentative influence on moral decisions is moderated by associative learning, but it is not yet clear whether such influence would be strong enough to deter cheating. Could religious environments affect moral behavior through the accumulation of arbitrary, subtle sensory cues associated with morality?

To answer this question, we suggest a novel approach to religious priming. We selected a stimulus that does not bear any inherent meaning by itself: instrumental music. While religions employ multiple symbols that could have been chosen, music is a widespread feature of religious environments that can be translated between different cultures (as opposed to specific symbols like Shiva lingam, Christian crosses, etc.). Moreover, numerous researchers have noted that music can play a significant role in social cohesion and cooperative behavior (Kirschner and Tomasello, 2010; Dunbar et al., 2012; Pearce et al., 2015; Lang et al., 2015b). It has been suggested that music can function as a proto-symbolic system that encompasses the structure of rituals, and that religious environments might have complex effects on people's social behavior (Alcorta and Sosis, 2005). Indeed, such a connection can be described as extra-musical meaning (Koelsch, 2011) or culturally enactive meaning (Cross and Morley, 2008), referring to explicit and conventional associations of music with real-world situations (e.g., anthems making people aware of their identity; Brown, 2000). This association may work similarly to the association with linguistic concepts. In an EEG study by Koelsch et al. (2004), participants were primed with sentences or musical excerpts that were semantically either related or unrelated to a word that followed. The authors recorded an event-related brain potential that is sensitive to a semantic fit (N400) and found no difference between sentences and musical excerpts. That is, when musical excerpts were semantically unrelated to the words that followed, the same error occurred as in the case of sentences. This result suggests that music can convey linguistic concepts and prime the meaning of a word (Koelsch, 2010). Such primes have been used, for instance, in a study of purchasing behavior, showing that when music is associated with information congruent with an advertised product, participants are more likely to be persuaded by the advertisement (North et al., 2004).

Besides the extra-musical meaning, musical stimuli carry information and messages that can elicit specific emotional responses, which in turn affect mood (Thompson et al., 2001) and morality judgments (Seidel and Prinz, 2013). For example, musical stimuli with positive valence decrease concerns regarding immoral messages and increase compliance with a request to harm others (Ziv et al., 2012; Ziv, 2015). Negatively valenced music, on the other hand, can increase participants' critical thinking (Sinclair et al., 2007). Furthermore, it has been shown that the tempo of musical stimuli can influence emotional arousal (Webster and Weir, 2005) and cognitive performance (Schellenberg, 2005; Schellenberg et al., 2007). However, we lack robust evidence showing that music influences participants' actual moral behavior (Ziv et al., 2012). And if it does, does this happen via the induction of specific emotions, through an association with conceptual complexes, or both?

The current study explored whether priming participants with instrumental religious music would decrease the rate of dishonest behavior. To isolate the effects of religious music, we designed three conditions: religious, secular, and control. After exposure to one of the three stimuli, participants' task was to solve a set of 20 matrices, and for each correctly solved matrix they received a monetary reward (Mazar et al., 2008). The number of correctly solved matrices was self-reported, thereby giving participants an opportunity to report dishonestly to increase their monetary reward and inflate their performance. We predicted that participants in the religious condition would behave less dishonestly than in the other two conditions. However, because instrumental religious music is not universally recognized as sacred (compared to religious concepts) and is thus less salient, we also expected that the effect of religious music would be moderated by participants' religiosity (congruent with the extra-musical meaning). That is, only religious participants would respond to this environmental cue that should activate an internalized behavioral schema (honesty). An additional supplementary hypothesis assumed the moderating effects of ritual participation frequency. The emotional characteristics, tempo, and impact of the presented stimuli were also assessed in order to test the hypothesis that music can affect decision-making through its affective component.

Addressing current debates on the generalizability of psychological studies (Henrich et al., 2010) and criticisms of religious priming literature and related meta-analytical research (Gomes and McCullough, 2015; van Elk et al., 2015; Shariff et al., 2016), we collected data from three different samples: a general population sample in Mauritius, and student population samples in the Czech Republic and the USA. By diversifying our participant pool, our goal was to control for possible culturally unique responses to religious primes. Despite demographic differences between these sites, we did not expect that priming with religious music should have different effects. We hypothesized that the learned link between religion and morality should work similarly in all sites. We were also interested to see whether general religiosity rates might play an important role in the effectiveness of religious primes, and we thus selected these three countries due to their different rates of general religiosity (Zuckerman, 2007; Gervais et al., under review).

## Materials and methods

### Participants

Data were collected from May 2014 to July 2015 in three sites: we recruited participants from the general Hindu population in Point aux Piments in Mauritius; a student population at Masaryk University in the Czech Republic; and a student population at Duke University, North Carolina, USA. Across the three sites, 254 participants were randomly assigned to one of three conditions: religious, secular, and control. Participants who previously took part in a similar experiment or showed suspicion about the experiment's goals were excluded from the final analysis (5 in Mauritius, 4 in the Czech Republic, and 13 in the USA). Overall, we tested 73 participants in Mauritius (20 females; M_age_ = 30.29, SD = 12.95); 78 participants in the Czech Republic (40 females; M_age_ = 24.05, SD = 3.69); and 81 in the USA (47 females; M_age_ = 22.74, SD = 3.77). Participants who did not fill out the parts of our questionnaire regarding musical stimuli (*n* = 12) were retained in the analysis of behavioral data, but were omitted from the analysis of musical stimuli. Participants were tested alone in rooms that contained only a chair, table, and computer. All materials, questionnaires, and consent forms were translated into the local languages (Mauritian Creole, Czech, and English). Informed consent was obtained from all participants. The study was approved by the Institutional Review Boards of Masaryk University, University of Connecticut, and Duke University.

### Material

In a double-blind design, participants were randomly assigned to one of three conditions defined by the type of stimulus they were exposed to: religious, secular, or control. Because we were specifically interested in the effects of music, none of the used musical excerpts contained any lyrics. All stimuli were of identical duration (2 min) and were administered via headphones in order to prevent interference from external noise. In the control condition, participants were exposed to white noise in order to control for possible effects of sound manipulation. While the control stimulus was the same across the three sites (Audio 7 in Supplementary Material), music in the religious and secular conditions was site-specific.

In Mauritius, we selected the appropriate religious music after consulting local religious experts, and comparable secular music after discussing with local research assistants. For the religious condition, we chose music that is often played during collective rituals in the local temple, and in particular during the annual religious festival of Thaipusam Kavadi (Audio 1 in Supplementary Material). This musical piece has dominant fast drums and a flute sound that is characteristic of the Kavadi ritual. For the secular condition we chose a popular Bollywood song (Mera Mahi Bada Sohna Hai—“Dhaai Akshar Prem Ke”; Audio 2 in Supplementary Material) that had similar sound and tempo to the music in the religious condition by sampling the first minute without any lyrics. This minute was looped in order to create a 2 min music sample.

In the Czech Republic and the USA, we pre-screened four Christian religious songs that are used during Catholic mass and four comparable secular songs. Participants from the Czech Republic and the USA rated them on 14 characteristics. These characteristics were combined into measures of stimuli's positivity, negativity, holiness, tempo, and impact (see Supplementary Material 1.1,1.2; and Tables S1, S2). In order to select secular stimuli that would be comparable with religious stimuli, we compared the most holy stimulus with the four pre-selected secular stimuli on the ratings of positivity, negativity, tempo, and impact, and selected the least different secular stimulus.

In the Czech Republic, 40 students from Masaryk University rated the eight selected stimuli. Since all of the religious songs had similar ratings of holiness (ranging from 4.28 to 4.43 out of 6), we chose the one that had the least mean difference in all other ratings with a secular song. Using this procedure, we selected an organ version of J. S. Bach's *Ave Maria* interpreted by Charles Gounod as the religious song (M_holy_ = 4.33, SD = 1.54; Audio 3 in Supplementary Material), and Tchaikovsky's *Romance for piano in F Minor, Op 5* as the secular song (Audio 4 in Supplementary Material). *Ave Maria* was performed on organs and Tchaikovsky's piece on piano, and both songs had similar tempos. The same procedure was used in the USA to select appropriate stimuli. We used Amazon Mechanical Turk to recruit 102 participants who rated the same songs as participants in the Czech Republic. For the Religious condition, we selected J.S. Bach's *BWV 147 Jesu joy of man's desiring*, which was rated as the most sacred song (M_holy_ = 2.94, SD = 2.10; Audio 5 in Supplementary Material). The most similar secular song was J.S. Bach's *BWV 140 Sleepers Wake* (Audio 6 in Supplementary Material). Although both songs were from the same composer, the religious one was performed on organs, while the secular one on piano.

### Procedure

Our experiment was conducted using Cogent 2000 developed by the Cogent 2000 team at the FIL and the ICN, and Cogent Graphics developed by John Romaya at the LON at the Wellcome Department of Imaging Neuroscience. Cogent 2000 was run as a Matlab Toolbox (2013a; MathWorks Inc., Massachusetts, USA). Participants were seated in individual rooms in front of a table with a computer, and a local research assistant explained that the purpose of the study was to investigate the effects of music on cognitive performance. Subsequently, the research assistant made sure that every participant understood the instructions (a practice item was presented) and instructed participants to keep their headphones on for the rest of the experiment. The research assistant then left the room, informing the participant that she or he would be working in the adjacent room and could be called when needed. The condition-specific musical stimulus played for 2 min, after which low-volume white noise was played for the rest of the experiment. This served to eliminate any possible disturbing noises.

**Figure 1:**
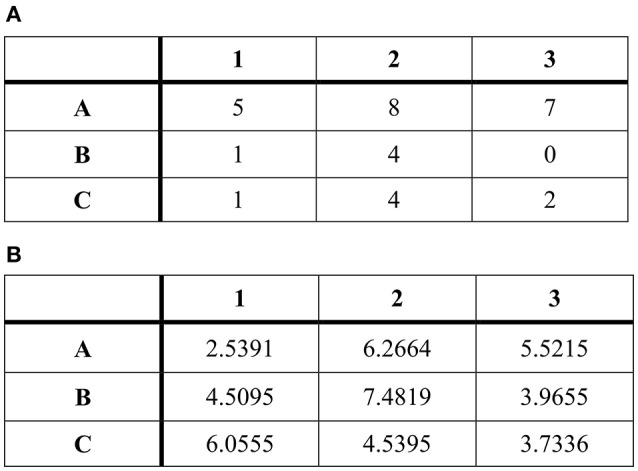
**A comparison of easy (A) and difficult matrices (B) used in the experiment**.

Once the music ended, a series of mathematical tasks appeared on the screen. The participants' task was to solve as many as they could out of a total of 20 given matrices (adapted from Mazar et al., 2008). Each matrix was presented on the screen in the form of a 3 × 3 table of numbers (see Figure 1). In each matrix, participants had to find two numbers that added up to 10 and remember their coordinates. There was always only one correct solution. Each matrix was presented for 15 s, after which participants had 6 s to think about the correct solution. Subsequently, the correct answer appeared on the screen for 3 s, and if it matched the solution that participants had in mind, they would make a mark on a prepared sheet. The prepared sheet contained one previously filled-out row, suggesting how many matrices the previous participant had successfully solved. Because almost no cheating was observed in a pilot that was run in the Czech Republic before the current study, we decided to encourage participants to cheat by suggesting that a previous participant cheated as well (Gino et al., 2009). Thus, the pre-filled row always contained eight marks. The matrix-solving task lasted 8 min in total.

After participants went through all 20 matrices they were instructed by the program to call the research assistant who then administered a post-study questionnaire and compensated participants based on their self-reported number of correctly solved matrices. The questionnaire assessed participants' religiosity, familiarity with the musical piece, ratings of the stimuli's positivity, negativity, holiness, tempo, and impact, and contained basic demographics (see Supplementary Material 1.4). We used the same approach to the construction of the stimuli's measures as during pre-screening the stimuli (see Supplementary Material 1.3). Debriefing was performed at the end of data collection.

For each correctly solved equation, participants were paid 5 MUR/10 CZK/0.5 USD. The maximum possible amount that participants could earn in each site was roughly equivalent to a budget restaurant meal. We did not control how many equations participants really solved correctly. However, in contrast to Mazar et al. (2008) who used the overall number of claimed matrices in their analyses, we approached the approximation of actual cheating in a more robust way. Using the raw untransformed data would introduce variability where, in theory, there should not be any. In other words, two participants might have solved three and five matrices respectively, seemingly showing variability in cheating while actually having chosen the same behavioral strategy (honesty). Whereas this problem could be addressed with a large sample, adding predictors at the level of an individual (e.g., religiosity) could bias a predictor's explanatory power. Furthermore, using the raw data would inflate the cheating scale and any differences in cheating would appear smaller than they were in reality.

To approximate the actual levels of cheating, we designed the experiment in such a way that most participants would solve five matrices. The first two equations were easy enough that everyone who passed the comprehension test should solve (adding up two numbers from 1 to 9), while the third matrix included numbers with three decimals, making it possible to solve in 15 s. In the rest of the matrices, the numbers contained 4 or 5 decimals, making it very difficult to solve in 15 s. However, participants could also guess the correct answer with a chance of 1:36 in each of the 17 remaining equations. According to the Bernoulli probability distribution, there is a 99% probability that a participant will not guess more than two solutions correctly. We thus assumed that participants who reported five or fewer solved equations were honest (i.e., possibly solving three and guessing two matrices).

To test this assumption, we recruited 112 participants from a student population at Masaryk University in the Czech Republic who were presented with the matrix task during a lecture in a large classroom. The matrices were projected on a wall and participants were instructed to write down answers (coordinates of two numbers adding up to 10) on a piece of paper. Participants who did not answer correctly any of the first two matrices without decimals were removed from the subsequent analysis (*n* = 12; such participants would not pass a comprehension test in our experiment), where we computed the average number of correctly solved matrices. Although the correspondence of pretest results with our assumption would not mean that we measured actual cheating, we believe that a low SD of pretest results together with a relatively large sample size provides sufficient precision for assessing the effects of our manipulation on participants' behavior.

### Data analysis

All data were analyzed in R (version 3.2.3, R Core Team, 2014). Since our data were bounded on the possible amount of dishonesty, we considered four different models: normal, normal censored, beta, and zero-inflated beta. While the untransformed data on cheating looked almost normally distributed (albeit leptokurtic; see Figure S2A), the values between 2 to 5 solved matrices mask the actual censoring. Since we considered five or fewer reported matrices as ethical behavior (see section Procedure), these values were collapsed to zero unfairly reported matrices. Thus, when this boundary was taken into account, histogram data showed a significant positive skew (see Figure S2B). Although the zero-inflation could be modeled by a censored model with normal distribution, the rest of the distribution (from the value of one and higher) was not normal either. We therefore considered a beta regression that uses a logit link to model means and variation in order to account for heteroscedasticity and skewness often present in bounded data (Stasinopoulos and Rigby, 2007; Cribari-Neto and Zeileis, 2010). To test whether a model with beta distribution would better fit the data, we transformed the number of claimed matrices to a percentage, with 15 being 100%—maximal dishonest behavior. Because our data also contained extreme values of 0 and 1 that are unacceptable for a beta regression model, we transformed the dependent variable using the formula (*y*′=*(y*·*(n* − *1)* + *0.5)/n*), where *y* is the transformed variable and *n* is the sample size (Smithson and Verkuilen, 2006). For the beta zero-inflated model, we used percentage data without transforming 0 and 1. A difference in Akaike Information Criterion (AIC) was used to compare models with different distributions (modeling only the intercept). From the four considered models, the one with beta distribution had significantly lower AIC than the other models (AIC_beta_ = −137.24, AIC_normal_ = 37.05). Thus, we used beta regression on the transformed data to model our dependent variable.

We fitted a beta regression model (Smithson and Verkuilen, 2006; Eskelson and Madsen, 2011) using the function *gamlss* (*gamlss* package; Stasinopoulos and Rigby, 2007). We built four sets of models. In the first set, we kept site as an independent factor in all models, controlling for differences between our sites. First, we modeled the main condition effect across all sites; subsequently, we added a Condition^*^Religiosity interaction to the model and compared it with a model that included a Condition^*^Ritual participation interaction; and lastly, we added possible covariates. In the first addition, age and sex were considered. The second addition comprised of the stimuli's positivity, negativity, tempo, and impact. In the second set of models, we analyzed condition effects and a Condition^*^Religiosity interaction at each site. In the third set, we considered covariates that could explain tentative differences between the sites. Namely, we looked at between-site differences in religiosity; ritual participation frequency; perceived holiness of the religious stimuli; perceived negative and positive emotional valence of the stimuli; and perceived tempo and impact of the stimuli. Finally, in the fourth set, we looked at the musical characteristics of the religious stimuli and their predictive power regarding unethical behavior in the religious condition. In all models with condition effects, we set the religious condition as a reference category for comparisons. That is, we were interested only in differences between the religious condition and the other two conditions. We assumed there should be no differences between the secular and control conditions. For the models of cheating that included site as a predictor, the USA was set as the reference category, but this choice was arbitrary. Specific between-site differences in overall cheating were not of interest in the current study—we used site only as a control for effects that were outside of our interest.

## Results

### Pretest

Results from the pretest confirmed our assumption that people on average solve five matrices (*n* = 100, *M* = 4.53, *SD* = 1.57). The minimum number of solved equations was two, while the maximum was nine. Although this range seems high at first, the frequency of participants that solved more than five matrices is exponentially decreasing (see Figure S1). We decided to set the cut-off at five as suggested by the mean number of solved matrices and Bernoulli probability distribution (see Procedure). In other words, we treated all participants in our experiment as behaving ethically if they reported five or fewer solved matrices. Six or more reported matrices were regarded as a scale of cheating.

### Manipulation check

An analysis of the perceived holiness of the stimuli across the three sites revealed a significant difference between conditions [*F*_(2, 217)_ = 20.63, *p* < 0.001]. Specifically, the religious condition had significantly higher ratings than the secular condition, and the control condition (ps < 0.001; see Table 1 for descriptive statistics). Looking at the emotional valence of the stimuli [*F*_(2, 217)_ = 4.64, *p* = 0.010], we found that the religious condition was perceived as significantly less negative than the control condition (*p* = 0.047). We did not observe any significant differences between the religious and secular conditions (*p* = 0.347). These results were replicated also for the positivity of stimuli [*F*_(2, 217)_ = 18.06, *p* < 0.001]: the religious stimuli were rated as significantly more positive compared to the control stimuli (*p* < 0.001), but not compared to the secular stimuli (*p* = 0.573). Similar results were obtained for our measures of tempo [*F*_(2, 217)_ = 6.90, *p* = 0.001] and impact [*F*_(2, 217)_ = 4.97, *p* = 0.008] of the stimuli. The religious stimuli were rated as significantly slower than the control stimuli (*p* = 0.001), but there was no difference between the religious and secular stimuli (*p* = 0.874). In terms of impact, the religious condition had significantly higher impact than the control condition (*p* = 0.002). The difference between the religious and secular condition was not significant (*p* = 0.219).

**Table 1:** **Descriptive statistics of dishonest behavior and musical-stimuli ratings**.

**Variable**	**Religious (*****n*** = **74)**				**Secular (*****n*** = **80)**				**Control (*****n*** = **78)**			
	**M**	**SD**	**CI**	***d***	**M**	**SD**	**CI**	***d***	**M**	**SD**	**CI**	***d***
% Claimed	30.27	27.35	24.04−36.05	–	31.50	24.41	26.37–36.63	0.05	34.96	27.71	28.81–41.12	0.17
Holiness	3.84	1.58	3.47−4.21	−	2.86	1.32	2.56−3.15	0.68	2.42	1.16	2.15−2.68	1.03
Negativity	2.28	0.92	2.10−2.49	−	2.13	0.80	1.95−2.31	0.17	2.59	1.09	2.34−2.84	0.31
Positivity	3.11	0.84	2.91−3.31	−	3.20	0.89	2.99−3.40	0.10	2.34	1.10	2.09−2.59	0.78
Tempo	2.73	0.96	2.50−2.95	−	2.76	0.83	2.57−2.94	0.03	3.23	0.96	3.01−3.45	0.52
Impact	3.26	1.13	3.01−3.53	−	3.01	1.28	2.73−3.30	0.21	2.63	1.63	2.34−2.91	0.53

*CI = 95% Confidence intervals. Cohen's d is the effect size of comparisons between the religious condition and the other conditions*.

### Dishonest behavior

**Figure 2:**
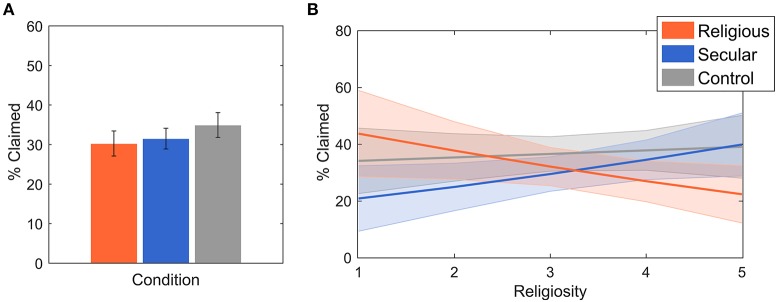
**(A)** The effects of different stimuli on the percent of matrices that were claimed as correctly solved above the expected levels with ±SEM. While controlling for the effects of site, there were no significant differences between conditions (see Table 2, Model 1). **(B)** Predicted values with 95% confidence intervals for the Condition^*^Religiosity interaction. The significantly different slopes suggest that religious participants cheated less upon being exposed to religious music (Table 2, Model 2).

To assess the amount of dishonest behavior among participants, we measured the percentage of matrices that were claimed as correctly solved and used beta regressions to estimate differences between predictors. We did not observe a significant difference between the religious and the secular (*p* = 0.44) and control conditions (*p* = 0.14). The estimates with significance levels from a beta regression are displayed in Table 2, Model 1 and plotted in Figure 2A. Looking at differences between the sites, participants in Mauritius claimed significantly more solved matrices than participants in the USA (*p* = 0.007), while participants in the Czech Republic claimed significantly fewer (*p* = 0.004; Table 2, Model 1). We observed a significant Condition^*^Religiosity interaction, with religious people cheating significantly less in the religious condition (*p* = 0.027). Compared to the religious condition, religiosity played a significantly smaller role in the secular (*p* = 0.026) and control conditions (*p* = 0.039; see Table 2, Model 2 and Figure 2B). That is, the more religious participants were, the less they cheated in the religious condition, while in the other two conditions religiosity did not significantly affect cheating. The model comprising a Condition^*^Ritual participation interaction suggested the same trend (for religious condition, *p* = 0.294), but the interaction was significant only for the secular condition (*p* = 0.011) and not for the control condition (*p* = 0.086; see Table 2, Model 3). From the considered covariates, only sex significantly improved the model fit. Aggregating across the three sites, on average males reported more matrices than females (*p* = 0.025; see Table 2, Model 4). There was no effect of perceived valence (*p*_negativity_ = 0.203; *p*_positivity_ = 0.335; *p*_tempo_ = 0.382; *p*_impact_ = 0.286) of the stimuli or of age (*p* = 0.847) on participants' behavior (Table 2, Model 5).

**Table 2:** **Estimates with SE from beta regressions for the percentage of matrices claimed as correct**.

**Predictor**	**Model 1**	**Model 2**	**Model 3**	**Model 4**	**Model 5**
Intercept	29.84 (3.61)^***^	30.32 (6.27)^***^	30.27 (6.19)^***^	31.18 (6.78)^***^	29.86 (6.49)^***^
Mauritius	11.32 (4.18)^**^	11.10 (4.44)^*^	9.28 (4.21) ^*^	7.93 (4.77)^Ϯ^	9.27 (5.19)^Ϯ^
Czech Republic	−9.61 (3.34)^**^	−9.63 (3.44)^**^	−9.38 (3.39)^*^	−10.50 (3.48)^**^	−9.75 (4.20)^*^
Secular	3.04 (3.93)	2.45 (3.92)	3.48 (3.95)	2.74 (3.97)	3.03 (3.93)
Control	5.99 (4.07)	5.40 (4.05)	6.01 (4.06)	6.19 (4.16)	7.60 (4.38)^Ϯ^
Religiosity		−5.31 (2.40)^*^		−4.97 (2.48)^*^	−4.97 (2.43)^*^
Secular^*^Religiosity		7.55 (3.37)^*^		7.54 (3.45)^*^	7.32 (3.37)^*^
Control^*^Religiosity		6.60 (3.18)^*^		6.49 (3.28)^*^	6.26 (3.18)^*^
Ritual			−1.55 (1.47)		
Secular^*^ Ritual			5.40 (2.12)^*^		
Control^*^ Ritual			3.54 (2.06)^Ϯ^		
Females vs. Males				7.90 (3.50)^*^	8.47 (3.48)^*^
Age				0.04 (0.20)	0.04 (0.20)
Positivity					−2.16 (2.24)
Negativity					−2.70 (2.12)
Tempo					−1.61 (1.84)
Impact					1.99 (1.86)
Cox-Snell R^2^	0.124	0.147	0.157	0.166	0.175

In all models, we control for the effects of site. The religious condition and the USA site were set as reference categories (intercept). The first model contains only the effects of condition (compared to the religious condition) while controlling for the effects of site. The second model includes a Condition^*^Religiosity interaction, describing the effects of religiosity on cheating in the religious condition. The two predictors specified as interactions (Secular^*^Religiosity and Control^*^Religiosity) are comparisons with this effect. Again, we control for site. The third model has an identical design to the second, only with a Condition^*^Ritual participation interaction. Since the effects of ritual participation on morality were not as strong as those of religiosity, we retained the latter factor for subsequent models. The fourth model contains site and condition effects, the significant interaction, and demographic covariates. The fifth model controls also for different characteristics of our musical stimuli.

Ϯ p < 0.1;

* p < 0.05;

** p < 0.01;

*** *p < 0.001*.

### Between-sites differences

**Figure 3:**
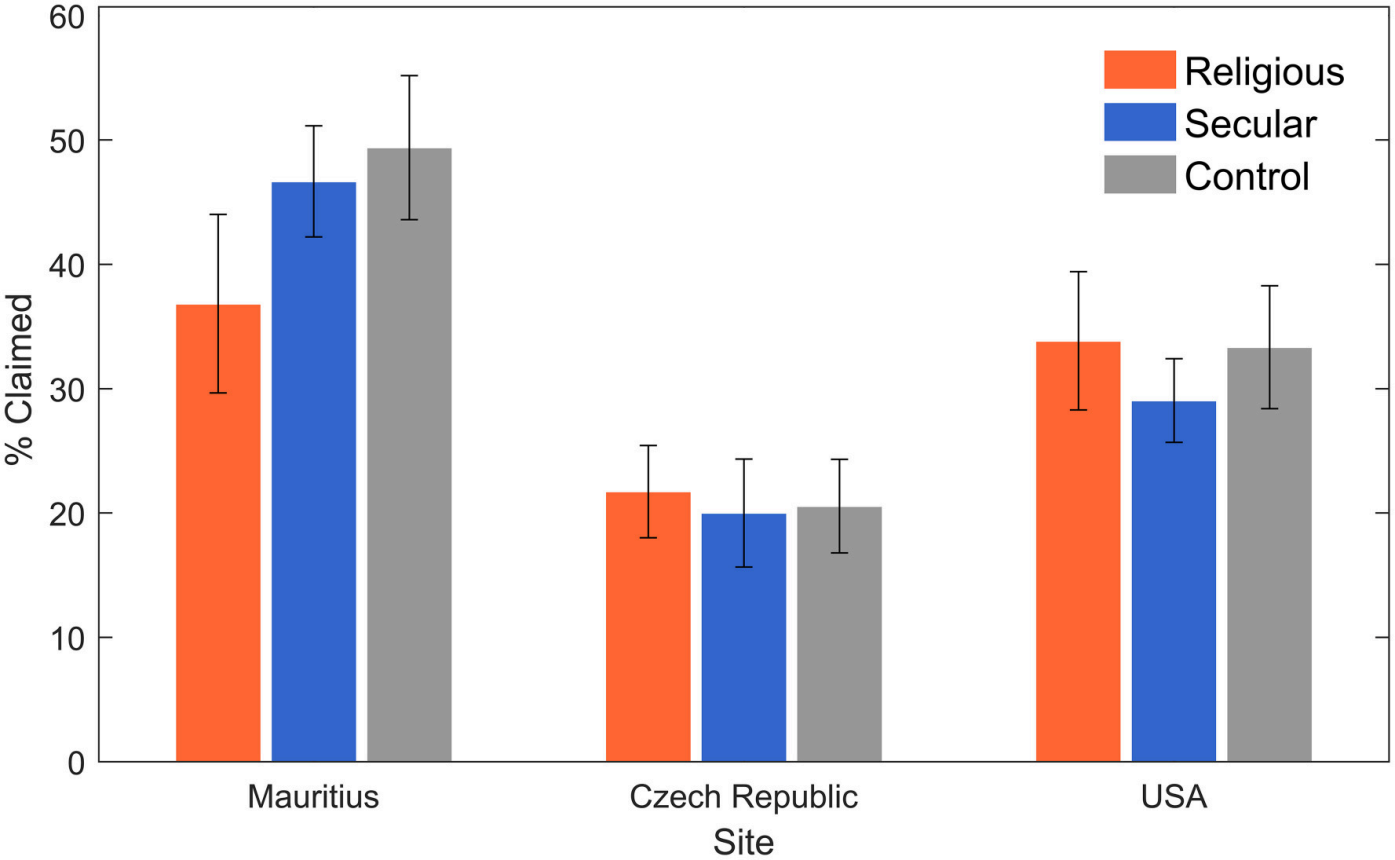
**The condition effect divided by site with ±SEM**. The only significant differences between conditions were found in Mauritius.

Focusing on the differences between our three sites (Mauritius, the Czech Republic, and the USA), we built separate models for the condition effects (see Table 3 and Figure 3 for descriptive statistics and Table 4 for model estimates). First, there was a significant difference between the religious condition and the other two conditions in Mauritius. Specifically, participants in the religious condition claimed a lower percentage of solved matrices than participants in the secular condition (*p* = 0.043) and participants in the control condition (*p* = 0.044). We did not observe a significant main effect of condition in the Czech Republic (religious vs. secular: *p* = 0.581; religious vs. control: *p* = 0.891). Likewise, the condition effect was not significant in the USA (religious vs. secular: *p* = 0.718; religious vs. control: *p* = 0.695). Looking at the Condition^*^Religiosity interactions, we observed a marginally significant interaction in the USA sample (Religiosity^*^Secular: *p* = 0.068; Religiosity^*^Control: *p* = 0.052), but this interaction did not replicate in the other sites (ps > 0.3; Table 4, Models B).

**Table 3:** **Descriptive statistics of between-sites differences in dishonest behavior (% Claimed)**.

	**Religious**					**Secular**					**Control**				
**Site**	***n***	**M**	**SD**	**CI**	***d***	***n***	**M**	**SD**	**CI**	***d***	***n***	**M**	**SD**	**CI**	***d***
Mauritius	21	36.83	32.91	22.75−50.90	−	25	46.67	22.36	37.90−55.43	0.35	27	49.83	30.11	38.02−60.74	0.40
Czech Rep.	27	21.73	19.27	14.46−28.30	−	27	20.00	22.57	11.49−28.51	0.08	24	20.56	18.43	13.18−27.93	0.06
USA	26	33.85	28.34	22.95−44.74	−	28	29.05	17.80	22.45−35.64	0.20	27	33.33	25.62	23.67−42.00	0.02

*CI = 95% Confidence intervals. Cohen's d is the effect size of comparisons between the religious condition and the other conditions*.

**Table 4:** **Estimates with SE from beta regressions for the percentage of matrices claimed as correct across our three sites**.

	**Mauritius**		**Czech Republic**		**USA**	
	**Model A**	**Model B**	**Model A**	**Model B**	**Model A**	**Model B**
Intercept	33.89 (5.57)^**^	35.49 (15.24)^**^	20.20 (3.57)^***^	21.70 (3.50) ^***^	33.82 (5.09)^**^	32.46 (9.78)^**^
Secular	16.66 (8.10)^*^	17.13 (11.33)	−2.54 (4.43)	−4.29 (4.46)	−3.17 (6.74)	0.49 (6.77)
Control	16.34 (7.95)^*^	13.83 (8.27)^Ϯ^	0.93 (4.88)	0.23 (4.83)	1.01 (7.03)	4.48 (6.97)
Religiosity		−4.72 (5.23)		−4.40 (3.22)		−5.04 (3.60)
Secular^*^Religiosity		2.41 (9.87)		1.58 (4.20)		9.91 (5.36)^Ϯ^
Control^*^Religiosity		7.58 (7.52)		−0.57 (3.81)		9.89 (5.01)^Ϯ^
Cox-Snell *R*^2^	0.071	0.085	0.008	0.077	0.005	0.068

Models A describe condition effects for the three sites: Mauritius, the Czech Republic, and the USA. Models B display a Condition^*^Religiosity interaction for each site. In all models, the religious condition was set as a reference category.

Ϯ p < 0.1;

* p < 0.05;

** p < 0.01;

*** *p < 0.001*.

**Figure 4:**
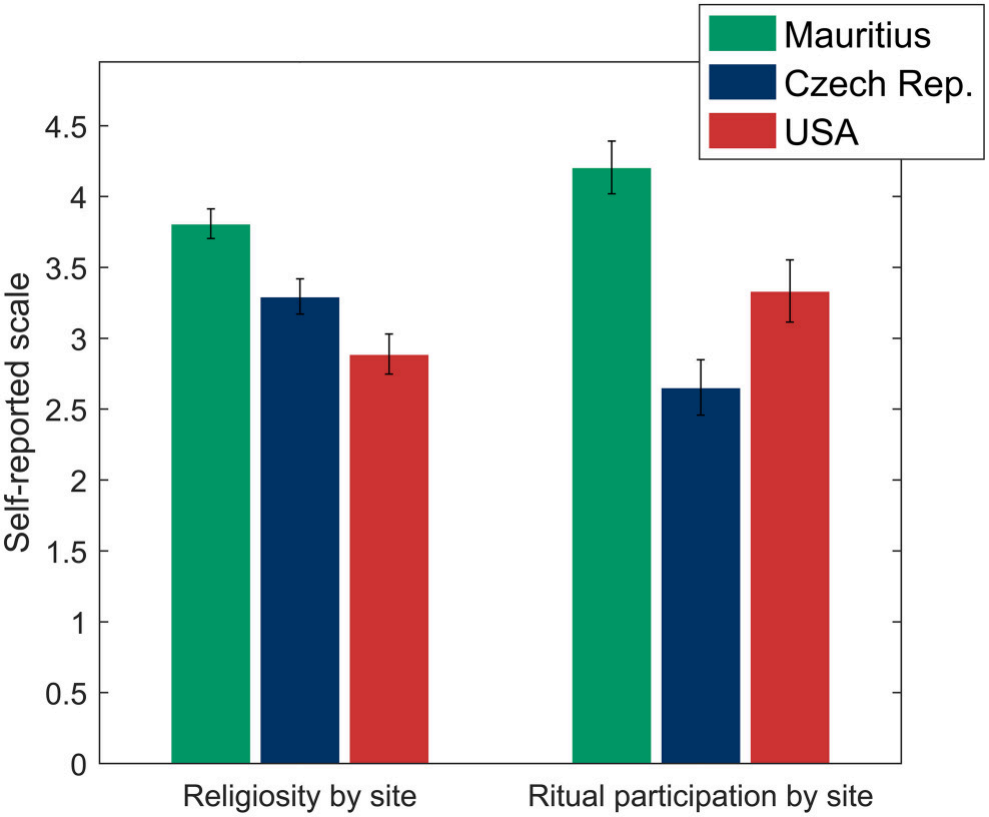
**Differences between sites in religiosity and ritual participation frequency with ±SEM**. Mauritian participants were significantly more religious and attended rituals more frequently than participants in the Czech Republic and the USA.

In order to better understand why the results from Mauritius differed from the other two sites, we used site as an independent variable (with Mauritius as the reference category) in predicting religiosity and ritual participation; and holiness, tempo, impact, and valence of the religious stimuli (see Table 5 for descriptive statistics). Mauritian participants reported being significantly more religious [*F*_(2, 229)_ = 13.31, *p* < 0.001] than those in the Czech Republic (*p* = 0.003) and the USA (*p* < 0.001). Similarly, participants in Mauritius reported significantly more frequent ritual participation [*F*_(2, 229)_ = 14.41, *p* < 0.001] compared to participants in the Czech Republic (*p* < 0.001) and the USA (*p* = 0.010). Religiosity and ritual participation are plotted in Figure 4.

**Table 5:** **Descriptive statistics of between-sites differences in religiosity and religious-stimuli ratings**.

	**Mauritius (*****n*** = **73)**				**Czech Republic (*****n*** = **78)**				**USA (*****n*** = **81)**			
**Variable**	**M**	**SD**	**CI**	***d***	**M**	**SD**	**CI**	***d***	**M**	**SD**	**CI**	***d***
Religiosity	3.81	0.89	3.60−4.01	−	3.30	1.09	3.05−3.54	0.51	2.89	1.28	2.61−3.17	0.84
Ritual participation	4.21	1.59	3.84−4.57	−	2.65	1.73	2.27−3.04	0.94	3.33	1.98	2.90−3.76	0.49
Holiness	3.41	2.00	2.46−4.36	−	3.85	1.32	3.35−4.35	0.26	4.12	1.51	3.54−4.69	0.40
Negativity	2.78	0.76	2.42−3.14	−	2.66	0.89	2.32−2.99	0.15	1.55	0.50	1.36−1.74	1.93
Positivity	2.59	0.69	2.26−2.92	−	3.55	0.73	3.27−3.83	1.35	2.99	0.83	2.68−3.31	0.53
Tempo	3.15	1.21	2.57−3.72	−	2.30	0.72	2.02−2.57	0.85	2.90	0.85	2.58−3.23	0.23
Impact	2.88	1.10	2.36−3.40	−	4.00	1.07	3.50−4.41	1.03	2.75	0.75	2.46−3.04	0.14

*CI = 95% Confidence intervals. Cohen's d is the effect size of comparisons between Mauritius and the other sites*.

There were no significant differences [*F*_(2, 67)_ = 1.03, *p* = 0.364] between Mauritius and the other sites in perceived holiness of the religious stimuli (Czech Rep.: *p* = 0.370; USA: *p* = 0.157). However, there were significant differences in perceived negativity of the religious stimuli [*F*_(2, 67)_ = 20.55, *p* < 0.001], with the Mauritian stimulus rated as significantly more negative compared to the USA (*p* < 0.001), but not to the Czech Republic (*p* = 0.592). Conversely, this pattern of significance was reversed for the positivity of the religious stimuli [*F*_(2, 67)_ = 8.83, *p* < 0.001], with the Mauritian stimulus being significantly less positive than the stimulus in the Czech Republic (*p* < 0.001) but not compared to the stimulus used in the USA (*p* = 0.093). Similar results were obtained for the tempo [*F*_(2, 67)_ = 5.37, *p* = 0.007] and impact [*F*_(2, 67)_ = 12.67, *p* < 0.001] of the religious stimuli. The Mauritian stimulus was rated as significantly faster than the stimulus used in the Czech Republic (*p* = 0.003), but there was no significant difference between Mauritius and the USA (*p* = 0.393). The Czech religious stimulus had a higher impact on participants compared to the Mauritian one (*p* < 0.001), but again, no significant difference was found between Mauritius and the USA (*p* = 0.664). In order to investigate whether these differences affected decision-making in the Religious condition, we built a model with the number of matrices claimed as a dependent variable, and the religious stimuli's characteristics as predictors. However, none of these characteristics explained any significant amount of variation in dishonest behavior in the religious condition (all ps > 0.29; see Table 6).

**Table 6:** **Estimates with SE from a beta regression for the percentage of matrices claimed as correct in the religious condition**.

Intercept	30.87 (5.89)^***^
Positivity	−0.54 (4.47)
Negativity	−0.70 (3.79)
Tempo	−3.23 (3.31)
Impact	−3.57 (3.37)
Cox-Snell *R*^2^	0.028

*Differences between sites in the characteristics of religious stimuli do not explain differences in the number of claimed matrices*.

## Discussion

We tested the hypothesis that non-verbal religious primes in the form of religious music would decrease dishonest behavior compared to secular music and white noise. Whereas it has been previously shown that religious words and complex religious contexts (e.g., a church environment) can increase participants' prosociality (Xygalatas, 2013), a possible effect of religion on deterring antisocial behavior was tested only by priming with religious words. We were interested in whether moral decision-making would be influenced by such a subtle cue as instrumental music. Participants in Mauritius, the Czech Republic, and the USA were given an opportunity to dishonestly inflate their performance in order to maximize their profit. This incentive to behave dishonestly was shown to be effective across all three sites. When collapsing all three sites together, we did not observe a significant effect of religious music on the rate of dishonest behavior. However, breaking down the condition effect by site revealed that religious music significantly decreased the incentive to cheat in Mauritius, but no such effect was observed in the other two sites. To test the hypothesis that the condition effect would be moderated by religiosity, we included a Condition^*^Religiosity interaction in our models. Religious music significantly reduced dishonest behavior in religious participants, while ritual participation frequency played a marginally significant role in the religious condition. Males displayed higher rates of dishonesty across the three conditions. Finally, participants' age and musical characteristics of the stimuli did not play a significant role. Together, these results offer a more nuanced interpretation of the influence of religious contexts on moral behavior.

It is important to acknowledge that the current study has several limitations. First, given the effect sizes for the differences between conditions at each site, we need to exert caution in interpreting the observed differences. While the collapsed sample across all sites is robust enough to detect medium effect sizes, the sample sizes at each site do not warrant generalizations due to low statistical power (Button et al., 2013). Furthermore, since the effect sizes of the differences between conditions in Mauritius are rather small (0.3 and 0.4), this finding needs to be further probed by future studies. Second, we did not collect exact data on actual cheating. While our procedure should secure confident estimates of unethical behavior, it is still possible that some participants correctly solved more than 5 matrices and vice versa. Similarly, some participants could feel that they found a correct answer and that the answer we provided was incorrect. Since the mathematical equations were computed under time-pressure, participants could make a small mistake without noticing and feel righteous to claim their answer as correct. However, given our overall sample size, such participants should constitute only a minimal portion of our sample. Third, since the musical stimuli were played before the mathematical task, their effects could be concealed by the time delay or the cognitive demands of the task. Perhaps if the stimuli were played during the whole experiment, the primes would be more salient and thus capable of influencing participants' behavior to a greater extent. Such a proposition needs further empirical testing. Fourth, the religiosity effect could have been mediated by some other mental process than by an association to normative behavior. For example, the thought of religion could have primed global processing, which has been previously shown to increase prosocial behavior (Mukherjee et al., 2014).

The lack of a main condition effect in the overall sample suggests that religious music might not always be salient enough to deter people from dishonest behavior. Although our religious stimuli were recognized as significantly more holy than the other two stimuli, honesty was only affected in one of three sites. A significantly lower amount of dishonest behavior in the religious condition was observed only in Mauritius, which points to the need for a more thorough understanding of differences between our sites. There are at least three possible interpretations: (a) this finding is a false positive; (b) participants in Mauritius were induced with different emotions that influenced their behavior; or (c) the association between religious music and normative behavior is stronger in Mauritius due to higher religiosity.

The observed difference between different conditions in Mauritius could have been caused by different characteristics of our religious stimuli. While we used organ music in the Czech Republic and the USA, the Mauritian religious stimulus had significantly higher tempo and dominant drums. A comparison of religious stimuli across sites revealed mixed results. The Mauritian religious music was perceived as significantly more negative than the religious stimulus in the USA, while there was no difference between Mauritius and the Czech Republic. We can speculate that, for example, Mauritian participants were more avoidant and critical due to higher negativity evoked by the religious stimulus and, consequently, avoided the cheating behavior. However, we find this interpretation unlikely because the perceived negativity of the stimuli was not significantly different between Mauritius and the Czech Republic. Similarly, differences between Mauritius and the other sites in positivity, tempo, and impact were always only between two sites, suggesting that no systematic differences were related to those properties. Furthermore, looking at the overall effects of musical characteristics on cheating rates, we did not observe any significant influence of these variables. This is in contrast with previous research which suggested that positively valenced music decreases moral concerns (Ziv et al., 2012). The lack of such effects might stem from the fact that the link between positive music and cheating was previously tested only by self-reports (Ziv et al., 2012). Alternatively, the cognitive demands of our task might have concealed any tentative subtle effects of musical characteristics.

The overall higher rates of self-reported religiosity and ritual participation frequency in Mauritius appear to be a more probable explanation of the behavioral differences between our sites. Religiosity is entrenched into Mauritian everyday life much more than in the other two sites, and might play a more important normative role (Xygalatas, 2013). This was confirmed by the significant differences in reported religiosity and frequency of ritual participation between Mauritius and the other two sites, and might indicate that higher religiosity could be associated with heightened sensitivity to religious cues (for similar results on prosocial behavior see Xygalatas et al., 2015). This interpretation is further supported by the significant Condition^*^Religiosity interaction. Collapsing all three sites, higher religiosity was associated with decreased rates of dishonest behavior in the religious condition. Although participants recognized our stimuli as religious, the less religious participants seemed to be unaffected. This result is in contrast with previous studies that showed no effect of religiosity on overall cheating rates (Randolph-Seng and Nielsen, 2007; Mazar et al., 2008; Aveyard, 2014). Our study thus offers new preliminary evidence on the role of religiosity, in congruence with the research on religious prosocial behavior (Shariff et al., 2016).

The fact that religiosity had a significant impact on dishonest behavior only in the religious condition supports the important role of religious situational factors in decision-making. We propose that dispositional religiosity does not affect participants' honesty to a large extent, unless it is activated by environmental sacred cues (Darley and Batson, 1973; Norenzayan and Shariff, 2008; Xygalatas, 2013; Xygalatas et al., 2015). While Mauritian participants reported significantly higher religiosity than participants at the other sites, the Mauritian cheating rates were significantly higher than those in the Czech Republic and the USA. Such a finding suggests that participants needed to be reminded of their religiosity in order for it to affect their moral decision-making. However, such a “reminder effect” is probably temporary (Malhotra, 2008) and confined only to religious participants. When religious cues are salient and general enough (e.g., the word God), they might affect non-religious participants, thus masking the effect of dispositional religiosity. But when subtle (as in the case of our study), these sacred cues only influence religious people who are more sensitive to them. This could also explain why studies that used linguistic primes (Randolph-Seng and Nielsen, 2007; Mazar et al., 2008) did not find a significant moderating effect of religiosity. Religious words are part of the standard cultural language toolbox and have stronger behavioral associations than specific religious symbols. For example, the Islamic call to prayer is a public, omnipresent cue that is directly associated with specific behaviors. As such, these cues are less ambiguous than music (Cross and Morley, 2008). Instrumental religious music, on the other hand, is generally less known, and associative learning is rather accomplished via communal socialization that reinforces the association of symbols with religion. Music is rarely associated with specific behavioral requirements, especially those regarding moral conduct. Behavioral schemas are thus not directly accessible to those who have not undergone religious socialization and do not participate in communal ritual gatherings (while they might be accessible to the majority of people through words). The fact that music is such a subtle cue can explain why we did not observe a significant Condition^*^Religiosity interaction in each of our sites. We would probably need larger sample sizes in order to show such an interactive effect.

The importance of ritual participation in the accessibility of behavioral schemas is further supported by a trend in the Condition^*^Ritual participation interaction. The fact that this trend did not reach statistical significance, however, suggests that ritual participation alone might not be enough to promote honest behavior (Mitkidis et al., 2014). It may reinforce the link between symbolic and behavioral schemas, but this link without an overarching religious worldview is probably a weak motivational force. Although participation in public rituals usually signals acceptance of religious norms (Rappaport, 1999), it is not necessarily tied to actual normative behavior and people can participate in these rituals for various reasons, for instance, reducing anxiety (Lang et al., 2015a), including no specific reason at all (Xygalatas, 2012). Such participants might be less inclined to follow normative schemas prescribed by their respective religions, especially if different behaviors have momentarily higher pay-offs (free-riding). Furthermore, ritual intensity may play an important role in the reinforcement of the link between symbol and behavior. High-intensity rituals are usually extremely arousing events (Xygalatas et al., 2013a,b), and as such might yield stronger affective bonds between symbols and conceptual complexes (Alcorta and Sosis, 2005). This might provide additional support for the suggested explanation of the differences in dishonest behavior between our sites. In Mauritius, we used music from the Kavadi ritual as the religious stimulus. The Kavadi is a high-intensity ritual that involves multiple body piercings, walking on nails, carrying heavy objects, and other forms of prolonged suffering. As such, it might be especially powerful in associating the musical stimulus with specific behavioral requirements and might have provided sufficient motivation for moral behavior that was not reached by religious stimuli that referred to less intense rituals in the other sites. This interpretation gains additional support by field experimental evidence that self-reported frequency of participation in the Kavadi ritual significantly predicted lower amounts of dishonest behavior in an economic game (Xygalatas et al., under review). We thus suggest that participation in high-intensity rituals might be effective in transforming behavioral requirements into symbols and as such be a powerful motivational force.

Our findings might be of importance for evolutionary models of music and its functions. Evolutionary theorists have disagreed on whether music is an evolutionary by-product or an adaptation. The by-product thesis argues that music parasitizes upon our evolved language abilities. In fact, Steven Pinker (1998) has dubbed music an “auditory cheesecake.” According to this view, our love for music is a by-product of specific cognitive-linguistic capacities, just like our love for junk food is a by-product of our adaptive need for fat, salt, and sugar. Others, however, point to the ubiquity of music across all cultures, as well as the fact that language and musical abilities are not strictly cognitively overlapping, and argue that music-making might have evolved as an adaptive trait (Fitch, 2006). For example, it might be an important tool for sexual selection, much like in birds (Miller, 2000), as suggested by the sex appeal of musical celebrities. Another important function might be related to an endorphin-based social binding mechanism (Dunbar et al., 2012) whereby music can function as social glue, a sort of “vocal grooming” (Weinstein et al., 2015). While these functions are not mutually exclusive, here we demonstrate that music may serve yet another function, that of representing norms and influencing behavioral schemas. We suggest that it does so via associative learning in communal gatherings where conceptual complexes are encoded in memory together with music. This link might be even stronger when norm-related words are included to create a song. Such songs can trigger outbursts of connotations, and thus function as a compact version of normative conceptual complexes, becoming effective vehicles for the transmission of social norms.

In summary, the current study provides preliminary support for the hypothesis that instrumental music can serve as a reminder of normative behavior, but only for participants who previously formed an association between religion and specific music. This result suggests that while socialization into group norms is crucial for ethical behavior, people need to be reminded of these norms to ensure an activation of normative behavioral schemes. In this respect, religion is a powerful institution that fosters normative behavior via shared rituals, repetitive songs and prayers, and other symbols that can act as associative triggers of ethical behavior. Further research should also investigate whether a combination of these triggers might possibly amplify their effects on participants' decision making. Likewise, using multiple sites within different cultural contexts in future research might help increase the reliability of priming studies and address the reproducibility crisis in psychological research (Open Science Collaboration, 2015).

## Author contributions

ML, PM, RK, and DX designed the study; ML, RK, AN, and LK collected the data; ML analyzed the data; ML, PM, AN, and DX wrote the paper.

### Conflict of interest statement

The authors declare that the research was conducted in the absence of any commercial or financial relationships that could be construed as a potential conflict of interest.
