# Supplementary material for: Music As a Sacred Cue? Effects of Religious Music on Moral Behavior
Source: Front Psychol. 2016 Jun 7;7:814. doi: 10.3389/fpsyg.2016.00814 (PMC4894891; doi:10.3389/fpsyg.2016.00814)
Supplement: Supplementary file 1 [file Presentation1.pdf]

Supplementary Material

Music as a Sacred Cue? Effects of Religious Music on Moral Behavior

Lang, Martin*[1,2], Mitkidis, Panagiotis[3,4,5], Kundt, Radek[2], Nichols, Aaron[3], Krajčíková, Lenka[6] & Xygalatas, Dimitris[1,2,4]

[1]*Department of Anthropology, University of Connecticut, Storrs, Connecticut, USA*
[2]*LEVYNA Laboratory for the Experimental Research of Religion, Department for the Study of Religions, Masaryk University, Brno, Czech Republic*
[3]*Center for Advanced Hindsight, Social Science Research Institute, Duke University, Durham, North Carolina, USA*
[4]*Interacting Minds Centre, Department of Culture and Society, Aarhus University, Aarhus, Denmark*
[5]*Interdisciplinary Centre for Organizational Architecture, Department of Management, Aarhus University, Aarhus, Denmark*
[6]*Department of Psychology, Faculty of Arts, Masaryk University, Brno, Czech Republic*

***Correspondence:**

Martin Lang

Department of Anthropology, University of Connecticut, 354 Mansfield Road, Storrs 06269, Connecticut, USA; martin.lang@uconn.edu

1. Supplementary Data

1.1 List of Musical Stimuli Used in Pre-screening

Religious stimuli:
J. S. Bach - Ave Maria (Gounod's interpretation)
Jan Zwart - Toccata Psalm 146
J. S. Bach - BWV 147 Jesu joy of man's desiring
J.S. Bach - BWV 29 We thank thee, God

Secular stimuli:
Max Richter - H In New England
P. I. Tchaikovsky - Romance for piano in F Minor, Op. 5
Yann Tiersen - Comptine d'Un Autre Été
J. S. Bach - BWV 140 Sleepers Awake

1.2 *Characteristics of Musical Excerpts Rated Pre-experiment:*

We adapted the Positive Affect Negative Affect Schedule (PANAS; Watson, Clark, & Tellegen, 1988) to measure positive and negative emotional characteristics of our musical stimuli (see section 1.4). We used Principal Component Analysis (PCA) with oblique rotation ("oblimin") to test if the selected characteristics load on positive (five items) and negative (four items) scales. The Bartlett's test of sphericity indicated that all items were sufficiently inter-correlated [χ^2 (36) = 3489.81, p < .001] and the Kaiser-Meyer-Olkin (KMO) test revealed sampling adequacy (MSA = .73). The number of factors was set to two because we expected to find one positive and one negative factor. Table S1 shows factor loadings. Both scales had sufficient Cronbach's α (positivity = .79, negativity = .68).

In addition to the positive and negative elements, we also included ratings of stimuli's holiness, tempo, and impact. The measure of holiness served to select a stimulus that would be associated with religion. The questions on the stimuli's tempo (How fast/slow was the stimulus?) were highly negatively correlated (r = -.62, p < .001), so the question "Slow" was reversed and both questions were combined into a measure of tempo. Similarly, we combined the measures of stimuli's deepness and strength (r = .51, p < .001) to assess stimuli's impact on participants. This measure served as a control for possible musical aspects that might prime participants similarly as religion but without the moralizing aspect.

1.3 *Characteristics of Musical Excerpts Rated Post-experiment:*

The same procedure as in the pre-experiment ratings was used to construct the measures of positivity, negativity, holiness, tempo, and impact for the post-experiment ratings. To construct the positivity and negativity measures, we used PCA with oblique rotation ("oblimin") and set the number of factors to two. The Bratlett's test was significant [χ^2 (36) = 687.91, p < .001] and the KMO score was adequate (MSA = .75). In contrast to the pre-experiment ratings, the item "Exciting" loaded highly on both factors. The items "Boring" and "Irritating" were strongly negatively loaded on the positivity factor and only weakly positively loaded on the negativity factor (see Table S2, Model 1). These results suggest that the scales obtained in our pre-

experiment ratings would not be replicated in the post-experiment ratings. We also combined the ratings of “Fast” and “Slow” into a measure of tempo, however, their correlation was not significant ($r = -.10$, $p = .125$). The only scale that replicated the findings from the pre-experiment screening was the impact scale consisting of items “Deep” and “Strong” ($r = .38$, $p < .001$).

In order to better understand why the post-experimental results differed from our pre-screening, we conducted a second analysis without the white-noise stimulus. Since rating white noise on tempo and emotional characteristics is rather ambivalent, we expected that scales for the religious and secular stimuli should better approximate the pre-screening scales. The factor loadings and Cronbach’s α are displayed in Table S2, Model 2. Indeed, loadings for the religious and secular stimuli resemble those of pre-screening with one exception: the item “Exciting” highly loaded on both factors. The correlation between “Slow” and “Fast” was significant ($r = -.18$, $p = .026$), as was the “Deep” and “Strong” correlation ($r = .31$, $p < .001$). Since these findings replicated the pre-screening findings, we created the measures of positivity (excluding the “Exciting” item; Cronbach’s α = .78), negativity (Cronbach’s α = .62), tempo, and impact. However, comparisons of the white-noise stimulus’ ratings with the religious and secular stimuli need to be interpreted with caution.

Table S1. Factor loadings of emotional characteristics used in pre-experimental ratings.

	Factor Loadings	
	Positivity	Negativity
Interesting	.85	
Pleasant	.84	
Exciting	.68	
Relaxing	.68	
Happy	.54	
Distressing		.85
Irritating		.68
Boring		.64
Sad		.63
Cronbach’s α	.79	.68

Table S2. Factor loadings of emotional characteristics used in post-experimental ratings. Model 1 shows loadings for all conditions, Model 2 includes only the religious and secular conditions.

	Factor Loadings Model 1		Factor Loadings Model 2	
	Positivity	Negativity	Positivity	Negativity
Interesting	.77		.79	
Pleasant	.76		.74	
Exciting	.46	.68	.54	.59
Relaxing	.71		.71	
Happy	.68		.34	
Distressing		.78		.83
Irritating	-.64	.27		.41
Boring	-.65	.27		.63
Sad		.73		.66
Cronbach's α	.78	.62	.78	.64

1.4 *Post-experiment Questionnaire*

1) Did you recognize the musical excerpt?

   a. Yes
   b. No
   c. I am not sure

2) Who was the author?

3) Did you perceive the sound as… (6-point scale)

   Profane o o o o o Holy

4) Please rate how much this song was…

   Not at all/A little/Moderately/Quite a bit/Extremely

   a. Sad
   b. Fast
   c. Boring
   d. Pleasant
   e. Happy
   f. Irritating
   g. Slow
   h. Exciting
   i. Deep
   j. Interesting
   k. Distressing
   l. Strong
   m. Relaxing

5) Are you a...

   a. Very religious person
   b. Religious person
   c. Neither religious/nor antireligious
   d. Rather secular person
   e. Not religious at all

6) Are you part of a church/religious organization?

   a. Yes
   b. No

7) If yes, what is your religion?

   a. Christian
   b. Jewish
   c. Muslim
   d. Buddhist
   e. Hindu
   f. Other

8) How often do you usually attend religious services/ceremonies?

   a. More than once per week
   b. Once per week
   c. Once per month
   d. Several times a year
   e. Once per year
   f. Not often at all
   g. Never

9) Are you

   a. Female
   b. Male

10) Please specify the year when you were born

2. Supplementary Figures

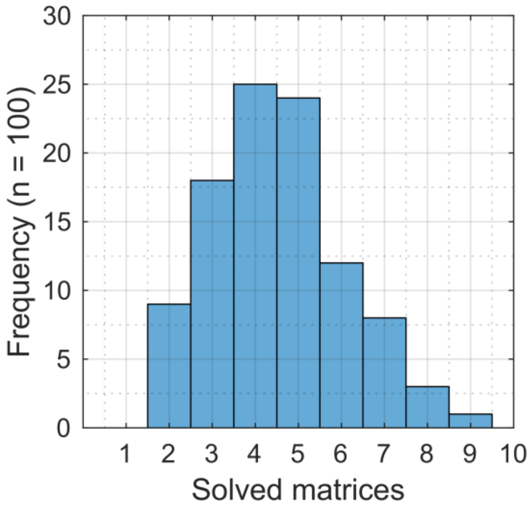

Figure S1. A histogram depicting the distribution of correctly solved matrices in the pre-experiment testing. The significant "jump" from five to six marks the border of ethical behavior in our experiment.

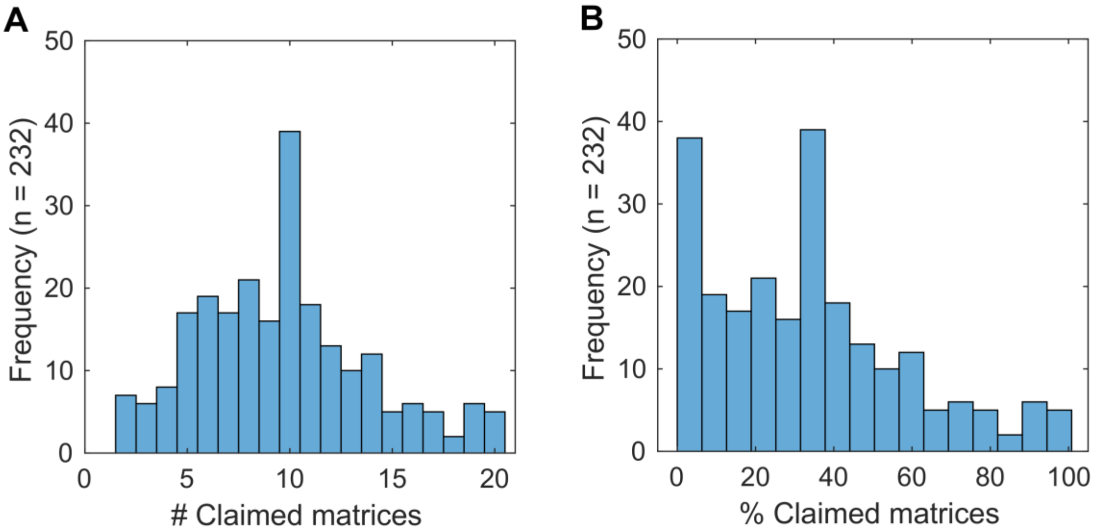

Figure S2. A. The distribution of the number of claimed matrices across our sites. **B.** The distribution of the percentage of unethically claimed matrices after collapsing the data for participants that solved between two and five matrices into 0% of claimed matrices.
